# The impact of a blend of *Pistacia atlantica* seed and canola oil compared with a blend of corn-canola oil with synthetic antioxidant and corn-canola oil without synthetic antioxidant on oxidative stress markers in patients with metabolic syndrome: protocol for a triple-blind, randomized, three-way cross-over clinical trial

**DOI:** 10.1186/s13063-023-07269-1

**Published:** 2023-07-24

**Authors:** Bahareh Sasanfar, Arezoo sadat Emrani, Faezeh Zademohammadi, Bita Forootani, Solaleh Emamgholipour, Sara Jambarsang, Sayyed Saeid Khayyatzadeh, Fatemeh Pourrajab, Seyed Ali Yasini Ardakani, Ahmad Esmaillzadeh, Amin Salehi-Abarghouei

**Affiliations:** 1 Research Center for Food Hygiene and Safety, School of Public Health, Shahid Sadoughi University of Medical Sciences, Yazd, Iran; 2 Department of Nutrition, School of Public Health, Shahid Sadoughi University of Medical Sciences, Yazd, Iran; 3 Cancer Research Center, Cancer Institute of Iran, Tehran University of Medical Sciences, Tehran, Iran; 4 Department of Clinical Biochemistry, School of Medicine, Tehran University of Medical Sciences, Tehran, Iran; 5 Department of Biostatistics and Epidemiology, School of Public Health, Shahid Sadoughi University of Medical Sciences, Yazd, Iran; 6 Department of Biochemistry and Molecular Biology, School of Medicine, Shahid Sadoughi University of Medical Sciences, Yazd, Iran; 7 Research center of food and confectionary, Yazd branch, Islamic Azad University, Yazd, Iran; 8 Department of Community Nutrition, School of Nutritional Sciences and Dietetics, Tehran University of Medical Sciences, Tehran University of Medical Sciences, Tehran, P.O. Box 14155-6117, Iran; 9 Obesity and Eating Habits Research Center, Endocrinology and Metabolism Molecular Cellular Sciences Institute, Tehran University of Medical Sciences, Tehran, Iran; 10 Yazd Cardiovascular Research Center, Non-communicable Diseases Research Institute, Shahid Sadoughi University of Medical Sciences, Yazd, Iran

**Keywords:** *Pistacia atlantica* oil, Corn oil, TBHQ, Metabolic syndrome, Antioxidant, Oxidative stress

## Abstract

**Background:**

Metabolic syndrome (MetS) is regarded as a complex metabolic disorder. Recently, the role of dietary antioxidants in the underlying pathogenesis and complications of MetS has come into focus. *Pistacia atlantica* oil is known as a high antioxidant oil which might improve the antioxidant status of dietary oils and also oxidative stress markers. On the other hand, tert-Butylhydroquinone (TBHQ) is an approved food-grade synthetic antioxidant that acts both as an inducer and inhibitor of carcinogenesis. The current trial will explore the possible effect of a blend of *Pistacia atlantica* seed-canola oils, corn-canola oils with TBHQ, and corn-canola oil without TBHQ on oxidative stress markers in patients with MetS.

**Methods:**

We will conduct a single-center, triple-blind, three-way randomized cross-over clinical trial (RCT) among 72 patients with MetS. After a 1-month run-in period, eligible participants will consume the intervention oils as their regularly consumed oils in a random order. Each intervention period will last 8 weeks separated by 4-week washout periods. Anthropometric indices, body composition, physical activity, blood pressure, and 24-h dietary food recall measurements will be assessed at the beginning and the end of each intervention period. The primary outcome will be oxidative stress markers including serum total antioxidant capacity, total oxidant status, malondialdehyde, nitric oxide, and the enzyme activity of myeloperoxidase, superoxide dismutase, catalase, glutathione peroxidase, and glutathione reductase. The secondary outcomes will be changes in MetS components including blood pressure, fasting blood glucose, triglyceride, high-density lipoprotein cholesterol, and anthropometric measurements.

**Discussion:**

*Pistacia atlantica* seed oil is high in antioxidants. An intervention with this oil could offer an option for oxidative stress prevention among patients with metabolic syndrome. The present clinical trial will be the first one assessing the impact of *Pistacia atlantica* oil on human oxidative stress markers.

**Trial registration:**

Iranian Registry of Clinical trials IRCT20130223012571N8. Registered on 4 March 2022.

## Strengths and limitations

- The first trial regarding the impact of *Pistacia atlantica* seeds oil on oxidative stress markers in humans.
- A triple-blind randomized controlled trial to assess the effects of three dietary vegetable oils.
- Bio-banking of blood components provides the opportunity to investigate the effect of dietary oils on different aspects of human health.
- The single-center design of the study limits the generalization of results to all populations.

## Introduction

*Pistacia atlantica* is a plant that grows in the Mediterranean region and the Middle East. The seeds are high in phytochemicals with antioxidant properties like α-pinene (20.8%), camphene (8.4%), myrcene (8.2%), and limonene (8%) [1, 2]. Also, its antimicrobial activity against both Gram-positive and negative bacteria was investigated [3]. In addition, it is proposed that *Pistacia atlantica* seed oil is a healthy oil with high amounts of omega-3 fatty acids [4].

Metabolic syndrome (MetS) is a set of conditions including obesity, dyslipidemia, increased blood pressure, and hyperglycemia [5]. This syndrome affects 33% of the adult population in the USA [6]. The condition affects about 18.7 million Iranians according to ATP ΙΙΙ definitions [7]. The pathogenesis of MetS is complex and unclear yet. It is proposed that this condition is stemmed from modern lifestyle and can increase the risk of type 2 diabetes, cardiovascular disease, cancer, and premature mortality [8–11]. There is ample evidence regarding the role of oxidative stress (OS), a condition in which there is an impaired balance between the production of reactive oxygen and nitrogen species and enzymatic and nonenzymatic antioxidant system defense capacity [12, 13], in insulin resistance and inflammation as the main pathogenic mechanisms of MetS [14]. Recent studies have demonstrated that diets rich in foods with high antioxidant content including fruits, vegetables, and whole grains, as well as diets low in animal fat, might improve the OS status [15, 16].

It was shown that *Pistacia atlantica* seed oil might help in preserving fatty acids that are prone to oxidation. For instance, *Pistacia atlantica* oil has been used as a natural alternative for stabilizing kilka fish oil [17]. Some animal studies have also explored the antioxidant effects of *Pistacia atlantica* on animals with colitis, peptic ulcer, diabetes, and MetS [1, 18–21]. A study on mice with diabetes showed significant improvements in lipid profile, OS, and inflammation markers after 3 weeks of intervention with *Pistacia atlantica* oil [18]. Another study on diabetic rats also illustrated a significant decrease in malondialdehyde (MDA) and increased GSH, GPx, CAT, and SOD after treatment with *Pistacia atlantica* oil [1]. On the other hand, tert-Butylhydroquinone (TBHQ), an approved food-grade synthetic phenolic antioxidant, is widely used as a preservative in many food products including vegetable oils [22, 23]. It is shown that TBHQ might have genotoxic effects; this is while other studies have revealed that it inhibits carcinogenesis [22, 24]. The permissible level of TBHQ in fats and oils is 0.02% or 200 mg/kg of fat or oil [25]. This level is low but many people consume appreciable amounts of phenolic antioxidants as food additives from dietary sources that typical consumption is likely to exceed the allowed daily intake (ADI, 0–0.2 mg/kg) [25].

To the best of our knowledge, no previous studies have examined the effects of *Pistacia atlantica* seed oil on oxidative stress in Humans. Furthermore, no study has tried to investigate if this oil improves oxidative stress when compared with conventional healthy oils available in the market in which TBHQ is used as the preservative. For this purpose, the present study was designed to examine the effect of a blend of *Pistacia atlantica* seed oil and canola oil compared with a blend of corn and canola oil with TBHQ (as a sample of conventional vegetable oils available in the market) and a blend of corn and canola oil without TBHQ (as a control oil) on oxidative stress markers in patients with MetS.

## Methods and analysis

### Study design and population

This trial is a single-center, triple-blind, randomized, superiority, three-way cross-over clinical trial (RCT) among 72 patients with MetS. Eligible participants will consume a blend of *Pistacia atlantica* seed (20%) and canola oil (80%), a blend of corn (20%) and canola oil (80%) with TBHQ (75 ppm), and a blend of corn (20%) and canola oil (20%) without TBHQ after a 4-week run-in period. We aim to replace the oils regularly used at home with intervention oils. As all family members will receive the intervention oils, all data will also be collected for the spouses of the participants. The intervention periods will last 8 weeks and will be separated with 4-week washout periods in which sunflower oil will be provided for the participants. Sunflower oil is highly consumed among Iranian families as the main cooking oil [26]. The trial will be conducted in the Nutrition and Food Security Research Center, Shahid Sadoughi University of medical science, Yazd, Iran. We used standard protocol items for randomized trials (SPIRIT) as a framework for reporting this study protocol [27].

### Recruitment and eligibility assessment

Recruitment will be taken place using advertisements and announcements at Endocrinology and Metabolism clinics and polyclinics of Shahid Sadoughi University of medical sciences, Yazd, Iran. The incidence of MetS in Yazd province is 56.1% [28]. The screening visit will be conducted by trained researchers for interested volunteers. During the first visit, the study procedure will be explained to participants, and informed consent will be obtained. Then, a questionnaire on socio-demographics and a 24-h physical activity recall will be completed for the participants. Also, anthropometric measurement, body composition, blood pressure measurement, laboratory assessment, and a 24-h food recall will be performed on the first visit by a trained nutritionist. Researchers will recommend the participants to maintain their physical activity all over the study period.

### Inclusion criteria

Adults aged 20 to 50 years with MetS [with at least three of these conditions: waist circumference > 102 cm in men and > 88 cm in women, systolic blood pressure > 130 mmHg or diastolic blood pressure > 85 mmHg, serum high-density lipoprotein (HDL) cholesterol < 40 mg/dl in men and < 50 mg/dl in women, serum triglyceride > 150 mg/dl, fasting blood glucose (FBG) ≥ 100 mg/dl] will be included in the present study [29].

### Exclusion criteria

Individuals reporting a history of cardiovascular disease, diabetes mellitus, any type of cancer, pancreatitis, liver disease, kidney disease, gastrointestinal disorders, history of changes in blood sugar and lipid-lowering pills over the past 3 months, changes in gastrointestinal medications less than 30 days before enrollment, weight changes over 3 kg during the 2 months prior to the study, allergy or intolerance to foods that will be used for the intervention, using alcohol consumption, using medication for hemostatic regulation (other than aspirin), taking systemic corticosteroids including androgen, phenytoin, erythromycin, taking drugs for COVID-19 including remdesivir, Interferon, actemra, favipiravir in the last 3 months, being pregnant, having intention to become pregnant, giving birth in the last 12 months, taking Omega-3 supplements more than 1 g/day, smoking more than 20 cigarettes a day, and those reluctance to follow the study protocol will be excluded from this study.

### Criteria for discontinuing or modifying allocated interventions

The intervention will be discontinued for participants in case of the occurrence of exclusion criteria and participants’ requests.

### Sample size

The sample size required to investigate and compare the outcomes of the study between the three groups was calculated by considering these items: due to the limited literature regarding the impact of *Pistacia atlantica* on antioxidant capacity in humans and citing the calculations from researcher’s own experiences about similar oils, the minimal effect size of 0.10–0.13 as a conservative average was considered for primary outcomes [30]. Also, a correlation between repeated measures as a conservative average was considered 0.5. The power and confidence interval for these calculations were considered as 0.80 and 0.95, respectively. Calculations were done using GPower 3.1.4.9. Therefore, the sample size for each treatment oil period was estimated to be 21 subjects; with taking a 10% of dropout, we required at least 24 people for each intervention period. Based on this calculation, 72 eligible participants will be entered into the current study. We will also calculate the power of this trial using a conservative estimate of a medium effect size.

Due to the limited literature regarding the impact of *Pistacia atlantica* on antioxidant capacity in humans and citing the expert’s opinion, the effect size [standardized mean difference (SMD)] was considered to be at least one. Based on this, considering the first type error of 5% and power of 80% and using the table of sample sizes calculated for factorial studies [31], the sample size in each group was estimated to be 21 subjects, which takes into account the 10% drop, requires at least 24 people in each intervention period. Based on this calculation, 72 eligible participants will be entered into the current study.

### Blinding, randomization, and allocation concealment

The three intervention oils will be provided in the same shape, color, appearance, and odor and will be packed in the same bottles. Bottles will be blinded for researchers and participants and will be labeled with three codes (A, B, C) by an independent researcher. The codes will not be released until after the statistical analyses; therefore, neither the study participants nor the personnel nor the statisticians will be aware of the intervention oils. We do not anticipate any requirement for unblinding but if required, the trial manager and data coordinator will have access to group allocations and any unblinding will be reported.

**Fig. 1:**
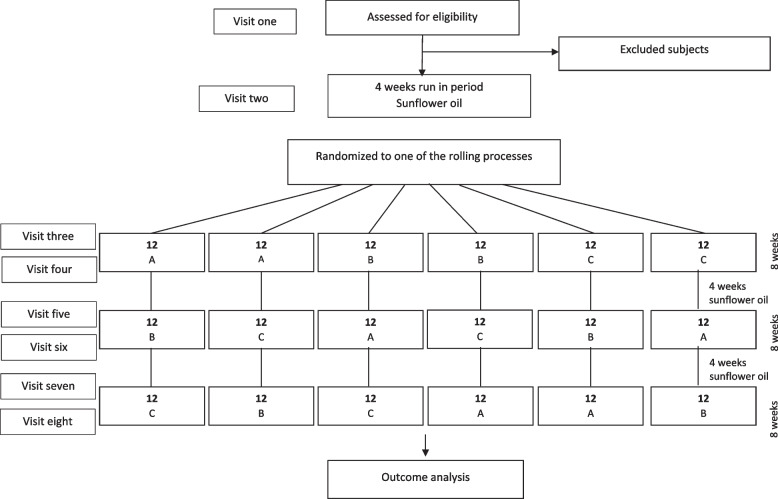
The study flow chart. Participants will be randomized to six rolling processes to receive one of the intervention oils labeled as A, B, and C

We will use a stratified block (with a block size of 6) randomization in which the stratification will be done based on sex. Then, the participants in each block will be randomly assigned to one of the 6 sequences of the rolling process to consume the three intervention oils during the study period (ABC, ACB, BAC, BCA, CAB, or CBA; Fig. 1). To ensure that all the subjects receive treatments in a balance and random order, computer-generated random numbers will be used. The allocation sequence and randomization will be conducted by an independent researcher and will be concealed from the researchers. This will be done by putting the randomly assigned sequence of interventions for each participant in sealed opaque envelopes.

### Intervention program

After the first visit, participants and their spouses will be entered into a run-in period for 4 weeks in which their regularly consumed oils will be replaced with sunflower oil. During the run-in period, the participants will be randomly assigned to one of the 6 rolling sequences (Fig. 1). The participants will be asked to replace the oils regularly used at home with the intervention oils. All measurements and blood sampling will be done at the beginning and the end of each intervention period, therefore, there will be 8 visits. The details of all measurements that will be conducted during each visit are provided in Table 1.

**Table 1:** Details of study visits

Measured variables	Beginning of study	Beginning of the run-in period	Beginning of phase 1	End of phase 1	Beginning of phase 2	End of phase 2	Beginning of phase 3	End of phase 3
	Visit 1	Visit 2	Visit 3	Visit 4	Visit 5	Visit 6	Visit 7	Visit 8
**Eligibility criteria assessment**	*							
**Medical history**	*							
**Informed consent**	*							
**Nutrition counseling**	*							
**Medication use**	*	*	*	*	*	*	*	*
**Physical activity**	*	*	*	*	*	*	*	*
**24-h dietary recall**	*	*	*	*	*	*	*	*
**Anthropometric measurement**	*		*	*	*	*	*	*
Weight	*		*	*	*	*	*	*
Height	*		*	*	*	*	*	*
Waist circumference	*		*	*	*	*	*	*
Hip circumference	*		*	*	*	*	*	*
**Body composition indices**			*	*	*	*	*	*
Body fat mass			*	*	*	*	*	*
Lean mass			*	*	*	*	*	*
Visceral fat			*	*	*	*	*	*
**Blood pressure**	*	*	*	*	*	*	*	*
**Blood sampling**			*	*	*	*	*	*
**Biochemical assessment**			*	*	*	*	*	*
Fasting blood glucose (FBS)	*		*	*	*	*	*	*
Triglyceride (TG)	*		*	*	*	*	*	*
High-density lipoprotein (HDL)	*		*	*	*	*	*	*
Total antioxidant capacity (TAC)			*	*	*	*	*	*
Total oxidant status (TOS)			*	*	*	*	*	*
Malondialdehyde (MDA)			*	*	*	*	*	*
Myeloperoxidase (MPO)			*	*	*	*	*	*
Superoxide dismutase (SOD)			*	*	*	*	*	*
Catalase (CAT)			*	*	*	*	*	*
Glutathione peroxidase (GPX)			*	*	*	*	*	*
Glutathione reductase (GR)			*	*	*	*	*	*
Nitric oxide (NO)			*	*	*	*	*	*
**Compliance**								
Three-day food recalls			*	*	*	*	*	*
Weight measurement of intervention oils			*	*	*	*	*	*

All anthropometric measurements, dietary and physical activity assessments, and blood sampling will be also performed for the participant’s spouses. Their blood samples will be kept in our biobank for future analyses and biochemical analyses will be also done on spouses.

### Executive group members

Five investigators will be involved in conducting the clinical trial (recruitment of study participants, visiting the participants, providing intervention oils, collecting blood samples, anthropometric measurements, dietary and physical activity assessments, data collection, and entry). The team will have regular weekly meetings with the principal investigator to ensure that the study will not deviate from the planned protocol. Monitoring and support of trial running will be done by monthly meetings with the trial’s steering committee [the principal investigators and four advisors].

### Chemical analysis of intervention oils

For exploring the fatty acid content of intervention oils and sunflower oil, gas chromatography with a flame ionizer detector (GC-FID) will be used before their delivery [32]. Also, the total antioxidant capacity of the oils will be assessed by the diphenyl-1-picrylhydrazyl free radical (DPPH) method [33]. The time between the production of intervention oils and delivery to participants will be 1 to 2 weeks and will be consumed in 8 weeks.

### Primary and secondary outcomes

The primary outcomes will be changes in serum total antioxidant capacity (TAC) and total oxidant status (TOS), malondialdehyde (MDA), nitric oxide (NO), the enzyme activity of myeloperoxidase (MPO), superoxide dismutase (SOD), catalase (CAT), glutathione peroxidase (GPX), and glutathione reductase (GR). We will use standard ELISA kits for the measurements. Mean difference ± SD values from the baseline will be reported. The time-point for intervention periods will be 8 weeks. The secondary outcomes will be changes in body weight and body composition measurements (waist circumference, hip circumference, total body fat mass, muscle mass, visceral fat mass), as well as, blood pressure, fasting serum glucose levels (FBG), systolic and diastolic blood pressure (SBP and DBP, respectively), serum triglyceride levels (TG), and high-density lipoprotein cholesterol (HDL-cholesterol).

### Anthropometry and blood pressure measurements

Height will be measured to the nearest 0.5 cm by a measuring tape mounted on the wall while the individual stands in a standing position without shoes. Waist circumferences will be measured to the nearest 1 cm using a non-stretchable measuring tape using a standard method [34]. Weight measurement and body composition analysis (% fat mass, % fat-free mass, and visceral fat) will be performed via a bioimpedance analyzer (InBody, model 770, Seoul, South Korea) while participants are with minimal clothes and not wearing shoes or stockings. Systolic and diastolic blood pressure (SBP and DBP) will be measured at the beginning and end of intervention periods after 5 min of rest when participants are in the sitting mode, for the right arm, using a sphygmomanometer (Riester Diplomat-presameter, Jungingen, Germany) (Table 1). All measurements will be performed 3 times at each visit (at the beginning and the end of each intervention phase) and their mean value will be recorded as the final value.

### Physical activity and dietary intake assessment

The participants will be asked to keep their physical activity level and dietary intake constant during the study. The participant’s physical activity will be assessed using 3-day physical activity records (2 weekdays and 1 day of the weekend). The 3-day physical activity records will be filled in 6 times during the study (at the beginning and end of each intervention phase) (Table 1). The recorded data will be converted into metabolic equivalent-min/day (MET-minute/day) using the updated version of the compendium of physical activities [35]. Three-day dietary recalls (2 weekdays and 1 day of the weekend) will be used to measure dietary nutrient intake, including macro and micro-nutrients, at the beginning and the end of each phase of the intervention.

### Blood samples

After an overnight fast (10–12 h), venous blood samples (15 mL) will be taken from participants between 7:00 and 9:30 a.m. at the beginning and end of each phase (Table 1). Then blood samples will be aliquoted to 2 serum samples, 1 plasma sample, 1 buffy coat sample, and 1 whole blood sample in DNase- and RNase-free 1-mL microtubes and will be stored at − 80 °C until analysis. Serum samples collected at the beginning and the end of each phase will be used for biochemical analyses.

### Laboratory assessment

Fasting serum glucose, serum TG, HDL-C, TAC, TOS, MDA, MPO, NO, the enzyme activity of SOD, CAT, GPX, and GR will be determined from serum samples using available standard kits.

### Compliance

The intervention oils will be provided for the participants and their families. We will ask participants to bring back intervention oil bottles provided in the previous visit. Then, these bottles will be weighed. Furthermore, 3-day dietary recalls will be used to assess the compliance of the participants to the intervention protocol.

### Assessment of medication use

Participants will be recommended to maintain their medication use during the study period. The researchers will also ask participants to inform them regarding the use and/or change of any medications during the study period since some drugs might have an impact on biochemical markers. To track their medication use, participants will be asked to bring all medications they consume during the intervention period at each visit. We will also ask them to report their usual medications (with their dosage) when contacted to assess the dietary intake data (Table 1).

### Assessment of adverse events and provisions for post-trial care

We anticipate that the intervention oils will have no harm or adverse effect on the participants. However, adverse events, if any, will be reviewed by the trial steering committee and reported to the ethics subcommittee for monitoring the clinical trials at Shahid Sadoughi University. After completing the intervention periods, the blood test results, assessment of food intake, and physical activity will be interpreted for the participants and necessary recommendations will be provided if needed.

### Data management

Data collection, coding, and entry will be done manually by the research staff for the study. Data will be kept by the principal investigator (ASA) of the research. Each study participant will be allotted a study code number and identifying information of study participants will be accessed by authorized personnel. Access to protocol, dataset, and statistical code will be available on reasonable request by the corresponding author.

### Statistical analyses

The statistical analysis will be performed using STATA software (version 14, TX, 77,845 USA). The normal distribution of the quantitative data will be determined using the Kolmogorov–Smirnov test, and the skewed variables will be normalized by transformation before analysis. The effects of treatment oils will be compared using the linear mixed method with the rolling process between-subject factors. Effect sizes will be adjusted by the potential confounders including age, sex, baseline BMI, the number of intervention oils consumed per subject, physical activity as metabolic equivalent-min/day, and the amount of calorie intake as covariates. Also, we will be performed subgroup analysis for sex. We will use the intention-to-treat method to handle protocol for those with non-adherence. No interim analysis is planned for this study because no early confirmatory conclusion of the effect of intervention oils can be drawn before the end of the study and enrollment of all participants. To control the carryover effect, clinically, an appropriate washout period will be considered (1 month). However, the carry-over effect will also be statistically tested through the mixed effect model. Any missing data will be handled using multiple imputations according to Harrell’s guidelines [36].

### Patient and public involvement statement

Study participants and the public were not involved in the research design. The primary outcomes will be discussed with the study participants via private meetings with the researchers after the analyses become completed.

### Ethics and monitoring

The ethical approval for conducting this study was obtained from the research ethics committee of Shahid Sadoughi University of Medical Sciences (2021, IR.SSU.SPH.REC.1400.167 (24 November 2022, https://ethics.research.ac.ir/PortalProposalList.php?code=IR.SSU.SPH.REC.1400.167). Also, the data monitoring committee, audit the consenting process, protocol adherence, and data collection, will be done by this ethics committee. Protocol amendments will be promptly reported to the research ethics committee of Shahid Sadoughi University of Medical Sciences. Also, the data monitoring committee, audit the consenting process, protocol adherence, and data collection, will be done by this ethics committee. Protocol amendments will be promptly reported to the research ethics committee of Shahid Sadoughi University of Medical Sciences. Data and safety monitoring committee (DSMC) will consist of 4 independent experts in clinical medicine, nutrition, biochemistry, and biostatistics. They will review the study protocol for any major concerns prior to implementation, regularly review and evaluate the accumulated study data for participant safety, study conduct, and progress, and make recommendations concerning the continuation, modification, or termination of the trial. They are independent of the study sponsor and have no competing interests. Bahareh Sasanfar will meet with the DSMC every 2 months and will present a written and verbal report. Additional meetings will be scheduled if warranted by the data or adverse events. The study protocol is also registered on 4 March 2022 in the Iranian Registry of Clinical trials (IRCT20130223012571N8, https://irct.ir/user/trial/60665/view). We will report protocol details and any amendments there. Informed consent will be obtained from all study participants. Participants will be allowed to leave the study without explanation and will not be compensated for participation. The study will be coordinated by the principal investigator at the Endocrinology and Metabolism clinics and polyclinics of Shahid Sadoughi University of medical sciences, Yazd, Iran. Study coordination, monitoring, data attainment and management, and statistical analysis will be performed by the principal investigator of the research. The International Committee of Medical Journal Editors (ICMJE) guidelines will be used for publishing the protocol and final manuscript. Professional writers will not be employed for writing.

### Dissemination

The results of this trial will be published in peer-reviewed scientific journals and presented at international academic meetings. We anticipate the findings will be of interest across broad fields like nutrition, endocrinology, MetS treatment, industry, and public health.

## Discussion

### Strengths and limitations of this study

The present clinical trial will be the first one assessing the impact of *Pistacia atlantica* oil on human oxidative stress markers. Previous investigations were done only on animals [1, 18–21]. This is a three-way randomized cross-over clinical trial in which subjects will act as their controls. This minimizes the effect of genetic polymorphisms, differences in lifestyle, and environment-related confounding variables on the observed effects. Furthermore, we will try to minimize the potential selection bias, confounding factor, and ascertainment bias by using standard methods for randomization, allocation concealment, and blinding.

The 8-week duration of intervention for each phase is long enough to investigate changes in total antioxidant capacity and total oxidant status. Additionally, with 72 participants and enough biological samples, our study enables researchers to examine the sex-stratified effect of the intervention oils and different markers with high statistical power. This sample size provides us with enough power to study possible gene-diet interactions in the future. Also, a unique feature of this study will be that we will compare novel oil blends with the usual oils that are available in the marketplace.

Medications used by participants will be recorded on each visiting day, then the investigators will be able to check the sensitivity of the observed effects to medication change by removing those who report a change in their medications. It is noteworthy that, all the procedures mentioned in the present study will be also conducted for the participants’ spouses, and thus, it is possible to assess the effect of the intervention oils in adults without MetS.

A limitation of this trial is that this is a single-center study which limits the generalization of results to all populations. Because of long-term intervention, the recruitment of study participants might be the challenging part of the study. To overcome this challenge, we will try to explain the benefits of participating in this study and the importance of study results for public health and industry. Endocrinologists will also help to increase the participation rate because people trust their physicians in Iran. As, in this trial, we aimed to substitute the common oils routinely consumed by participants with intervention oils; therefore, it will be not possible to calculate the exact amount of oils consumed by each person. However, we will try to resolve this problem by asking the participants to report the amount of oil consumed as tablespoons in their food recalls, as well as, the bottle oils will be weighed before and after consumption of each phase.

### Future investigations

The researchers are planning to explore the effect of the intervention oils on markers of glucose control including fasting serum insulin, cardiometabolic markers including total cholesterol, low-density lipoproteins (LDL-C), oxidized LDL, Apo A, Apo B, Lipoprotein (a) [LP (a)], liver enzymes [alanine aminotransferase(ALT), aspartate transaminase (AST), gamma-glutamyl transferase (GGT), alkaline phosphatase (ALP)], blood markers of kidney function [blood urea nitrogen (BUN), serum creatinine, and glomerular filtration rate (GFR)], and inflammatory markers [high sensitivity C-reactive protein (hs-CRP), interleukin-6 (IL-6), tumor necrosis factor-alpha (TNF-α)] in participants with MetS and their spouses in the near feature. Also, the current study provides a good medium for investigators to examine the possible interaction effect of gene polymorphisms and intervention oils on inflammatory and cardiometabolic markers. In this regard, investigators will explain the study objectives for the participants and informed consents will be obtained. The investigators will welcome possible collaborations with interested scientists and novel hypotheses that could be checked using the available samples and data obtained in the current study.

## Conclusion

As the relation between total antioxidant capacity and several diseases was confirmed by previous studies, we will explore the effect of substitution of regular oil consumed in a household with a blend of *Pistacia atlantica* seed and canola oil, with a blend of corn and canola oil with TBHQ and a blend of corn and canola oil without TBHQ on oxidative stress markers in participants with MetS and their spouses. In this three-way, triple-blind, clinical trial, the bio-banking of blood components will be done for study participants and their spouses will provide the opportunity to investigate the effect of dietary oils on different aspects of human health.

### Name and contact information for the trial sponsor

Shahid Sadoughi University of Medical Sciences (http://www.ssu.ac.ir) and the Neshatavar food industry (Datis Corporation; http://www.neshatavar.com/?l=EN).

### Trial status

The trial recruitment will begin on 22 May 2022. All trial participants should have finished the interventions up to 20 March 2023.

### Protocol version

Version 1.1, January 2023.

## Data Availability

The data of the present study will be fully available to the corresponding author. As data was approved to be used for the current analysis, only, it will not be available to the public. Any data required to support the protocol can be supplied on request.

## Abbreviations

ALP: Alkaline phosphatase
ALT: Alanine aminotransferase
AST: Aspartate transaminase
BUN: Blood urea nitrogen
CAT: Catalase
DBP: Diastolic blood pressure
DPPH: Diphenyl-1-picrylhydrazyl free radical
FBG: Fasting blood glucose
GC-FID: Gas chromatography with a flame ionizer detector
GFR: Glomerular filtration rate
GGT: Gamma-glutamyl transferase
GPx: Glutathione peroxidase
GR: Glutathione reductase
HDL: High-density lipoprotein
hs-CRP: High sensitivity C-reactive protein
IL-6: Interleukin-6
LDL-C: Low-density lipoproteins
LP (a): Lipoprotein (a)
MDA: Malondialdehyde
MetS: Metabolic syndrome
MPO: Myeloperoxidase
NO: Nitric oxide
OS: Oxidative stress
RCT: Randomized cross over clinical trial
SBP: Systolic blood pressure
SOD: Superoxide dismutase
SPIRIT: Standard Protocol Items for Randomized Trials
TAC: Total antioxidant capacity
TBHQ: Tert-Butylhydroquinone
TG: Triglyceride
TOS: Total oxidant status
TNF-α: Tumor necrosis factor alpha
